# Transgenic *Eimeria magna* Pérard, 1925 Displays Similar Parasitological Properties to the Wild-type Strain and Induces an Exogenous Protein-Specific Immune Response in Rabbits (*Oryctolagus cuniculus* L.)

**DOI:** 10.3389/fimmu.2017.00002

**Published:** 2017-01-23

**Authors:** Geru Tao, Tuanyuan Shi, Xinming Tang, Donald W. Duszynski, Yunzhou Wang, Chao Li, Jingxia Suo, Xiuling Tian, Xianyong Liu, Xun Suo

**Affiliations:** ^1^State Key Laboratory of Agrobiotechnology, China Agricultural University, Beijing, China; ^2^National Animal Protozoa Laboratory & College of Veterinary Medicine, China Agricultural University, Beijing, China; ^3^Department of Animal Parasitology, Institute of Animal Husbandry and Veterinary Medicine, Zhejiang Academy of Agricultural Science, Hangzhou, China; ^4^Department of Biology, University of New Mexico, Albuquerque, NM, USA

**Keywords:** rabbit coccidia, transgenic coccidia, *Eimeria magna*, life cycle, immunity

## Abstract

Rabbit coccidiosis causes great economic losses to world rabbitries. Little work has been done considering genetic manipulation on the etiological agents, rabbit *Eimeria* spp. In this study, we constructed a transgenic line of *Eimeria magna* (*EmagER*) expressing enhanced yellow fluorescent protein (EYFP) and red fluorescent protein (RFP) using regulatory sequences of *Eimeria tenella* and *Toxoplasma gondii*. We observed the life cycle of *EmagER* and confirmed that the transgenic parasites express exogenous proteins targeted to different cellular compartments throughout the entire life cycle. EYFP was expressed mainly in the nucleus and RFP both in the nucleus and cytoplasm. Then, coccidia-free, laboratory-reared 40-day-old rabbits were primarily infected with either *EmagER* or wild-type strain oocysts and challenged with the wild-type strain. *EmagER* showed similar reproductivity and immunogenicity to the wild-type strain. Finally, we examined the foreign protein-specific immune response elicited by *EmagER*. Rabbits were immunized with either transgenic or wild-type oocysts. Immune response against parasite-soluble antigen, EYFP and RFP in spleen, and mesenteric lymph nodes were detected by quantitative real-time PCR. The relative expression level of IFN-γ, IL-2, and TNF-α were higher in *EmagER*-immunized rabbits than wild-type parasites-immunized rabbits after stimulation with EYFP and RFP. Our study confirmed that a specific immune response was induced by the exogenous protein expressed by *EmagER* and favored future studies on application of transgenic rabbit coccidia as recombinant vaccine vectors.

## Introduction

*Eimeria* spp. are obligate intracellular parasites that infect epithelial cells of a particularly wide range of vertebrate species (1). Severe infections with *Eimeria* spp. can result in a serious disease, coccidiosis, which causes huge economic losses in two primary food animal industries, namely poultry and rabbitry (2–4).

Genetic manipulation of chicken *Eimeria* spp. has been an active and ongoing area of research for almost two decades (5) because it can be a strong tool toward understanding gene function and intimate interaction between these parasites and their hosts. To date, several transgenic chicken *Eimeria* lines have been developed. Besides *Eimeria tenella* (6), successful stable transfection of *Eimeria mitis* also was developed. Recently, Qin et al. (7) demonstrated that enhanced yellow fluorescent protein (EYFP) expressed by transgenic *E. mitis* contributed to a more detailed observation of this parasite’s endogenous stages. Not only for exploration on parasites’ biological characteristics, transgenic *Eimeria* parasites have great potential to be utilized as live recombinant vaccine vectors. Previous studies demonstrated that the exogenous protein expressed by transgenic parasites stimulate specific immune responses in the host. One study (8) showed that immunization with a transgenic *E. mitis* line expressing chicken IL-2 stimulated an enhanced cellular immune response against it. In addition, a transgenic line of *E. tenella* expressing *Toxoplasma gondii* SAG1 protein (TgSAG1) elicits protective immunity against *T. gondii* in chicken and mice (9). All these reports reinforced the broad prospect of transgenesis of *Eimeria* spp.

Similar work with rabbit *Eimeria* spp., however, lags far behind what we know about chicken coccidia. *Eimeria magna* is a common species of rabbit coccidia often found in great numbers on rabbit farms (11). It locates in the small intestine and produces a moderate pathogenicity and immunogenic response in rabbits (12, 13). In this study, we constructed a transgenic line of *E. magna* (*EmagER*) expressing double reporter genes, EYFP (7) and red fluorescent protein (RFP). Further investigations of its life cycle, reproductivity, and immunogenicity were conducted to provide more information on transgenesis in rabbit coccidia and evaluate the potential capacity as vaccine vector.

## Materials and Methods

### Ethics Statement

All experimental procedures were approved by the China Agricultural University Animal Ethics Committee (certified by Beijing Laboratory Animal employee, ID: 1114120800096), and due attention was paid to the welfare of the animals.

The rabbits were reared under stress-free environment, eliminating strong light and noise, with one rabbit per cage. Physical condition was monitored every day during all experimental procedures. Euthanasia was performed with an intracardiac pentobarbital overdose in accordance with the experiment design (14).

### Plasmid, Parasites, and Animals

**Figure 1:**
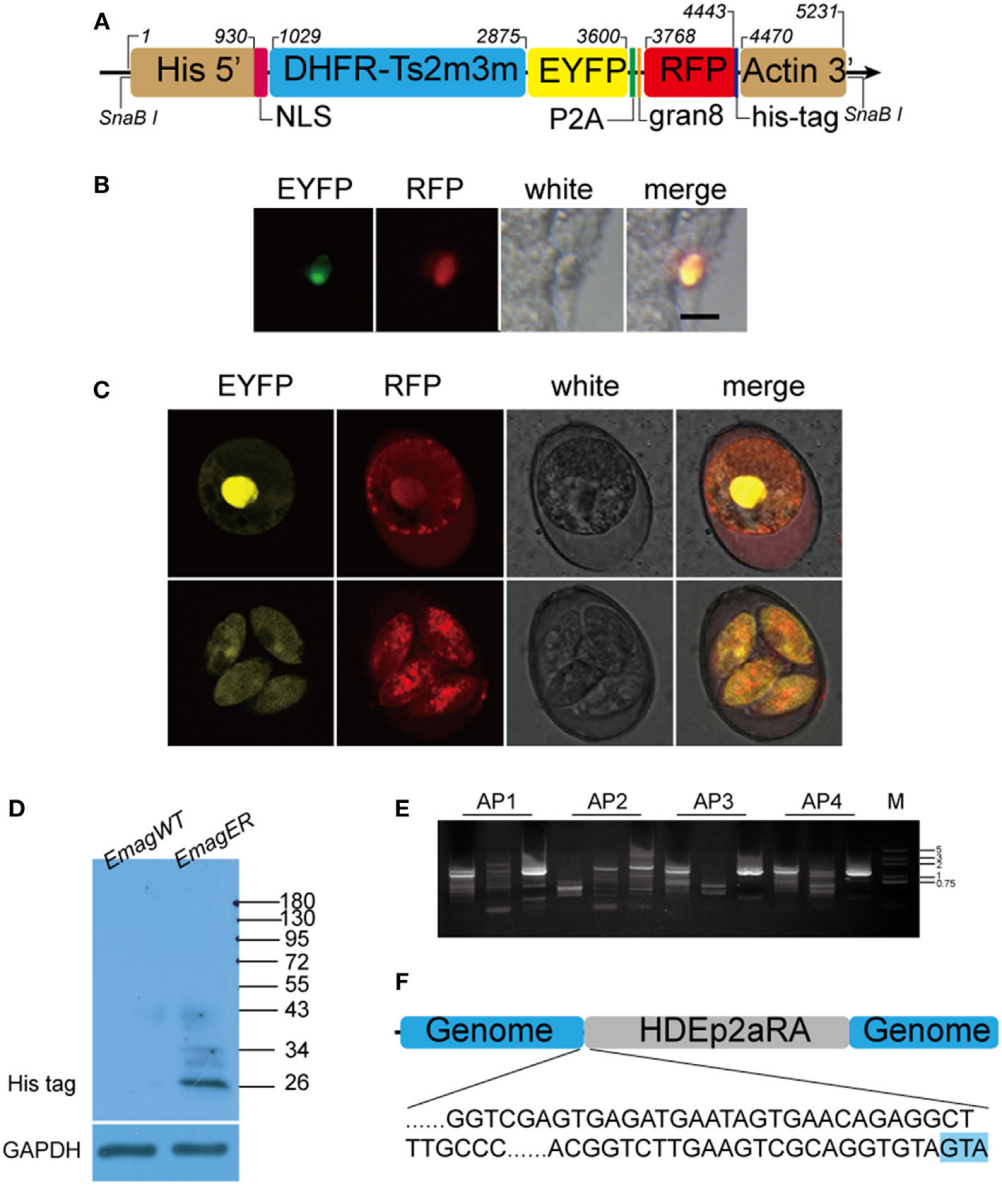
**Construction and identification of transgenic *Eimeria magna*-expressing enhanced yellow fluorescent protein (EYFP) and red fluorescent protein (RFP) (*EmagER*)**. **(A)** Schematic diagram of transfected linearized plasmid HDEp2aRA. DHFR-Ts2m3m fused with EYFP and RFP were inserted between promoter of histone 4-NLS and 3′ UTR of actin from *Eimeria tenella*. NLS, nuclear localization signal of histone 4; P2A, porcine teschovirus-1 2A peptide; gran8, signal sequence of *Toxoplasma gondii* dense granule 8. **(B)** Transient transfection of *E. magna* sporozoites in MDBK culture. Freshly purified sporozoites were electroporated with 15 µg *p*HDEp2aRA and inoculated into MDBK cell culture. Fluorescent sporozoites were observed 20 h post inoculation. Bar = 10 μm. **(C)** Unsporulated and sporulated oocysts of *EmagER*. EYFP was mainly located in the nucleus and RFP in the nucleus and the cytoplasm. **(D)** Western blot identifying P2A function in *EmagER*. Soluble abstract of *EmagWT* and *EmagER* were subjected to SDS-PAGE and Western blot using mAb against His-tag. A polyclonal antibody against GAPDH was included as a loading control. **(E,F)** Genome walking analysis identified the 5′ insertion site of the transfected plasmid. GTA in blue shading indicated cleaved linearization site (*Sna*BI).

Plasmid used in the transfection, *p*HDEp2aRA, is a single expression cassette where DHFR-Ts2m3m, a pyrimethamine resistance gene from *T. gondii*, EYFP, and RFP were inserted between histone 4 promoter with its nuclear localization signal (90 bp) (15) and 3′ untranslated region of actin of *E. tenella*. A porcine teschovirus-1 2A peptide (P2A) (66 bp), which was shown to be able to cleave two contiguous proteins (10, 16), was added between EYFP and RFP. Signal sequence of dense granule 8 (gran8) from *T. gondii* (84 bp) (17) was added to regulate secretion of RFP, which was followed by a His-tag (Figure 1A).

The wild-type *E. magna* (*EmagWT*) was originally isolated from Hebei province, China. Newly collected parasites were purified by flotation with saturated salt water followed by washing with sterilized water and incubation with 5% sodium hypochlorite to eliminate most of the bacterial contamination. Purified parasites, both wild-type and transgenic strain, were propagated in coccidia-free rabbits and maintained in 2.5% w/v potassium dichromate at 4°C according to a standard protocol (18). For propagation of the parasites, 200 sporulated oocysts per rabbit were orally inoculated. Oocysts were collected from feces of 7–10 days post inoculation (dpi). Moreover, fluorescent sporulated oocysts of the transgenic strain screened by the fluorescent-activated cell sorting (FACS) were used in propagation or other assays.

All animals used in this study were obtained from Xinglong Laboratory Animal Breeding Center. To avoid coccidial contamination, 3-week-old New Zealand white rabbits were weaned and reared by bottle-feeding of powdered milk under coccidia-free condition away from their mothers till 4 weeks old. Also, 35- to 40-day-old rabbits for parasite propagation and other assays were kept in isolators and fed with coccidia-free pellet feed and water *ad libitum*.

### Transfection of *E. Magna*, Selection, and Propagation of *EmagER*

Transfection of *E. magna* sporozoites was conducted according to an established protocol (6). Briefly, 2 × 10^7^ purified sporozoites were electroporated using a nucleofector (Program U-033, AMAXA, Switzerland) by a restriction enzyme-mediated method in a system containing 100 µl cytomix buffer, 15 µg linearized DNA, and 250 UI *Sna*BI restriction enzyme (15). Sporozoites were resuspended in DMEM and inoculated to MDBK culture for *in vitro* transient transfection or injected into duodenum of a 4-week-old rabbit in a laparotomy for *in vivo* stable transfection. In stable transfection, oocysts from feces of 7–10 dpi were collected as described above. Screening of the transgenic oocysts was conducted by FACS (MoFlo Cell Sorter, Dako-Cytomation, Fort Collins, CO, USA) and addition of 150 mg/kg pyrimethamine in the rabbit pellet as a drug selection was performed.

### Genomic and Expression Analysis of Exogenous Genes of *EmagER*

Integration site and expression of the exogenous DNA were investigated by genome walking analysis and Western blot. First, the flanking sequences of the 5′ integration site were identified using a genome walking kit (Takara, China). *EmagER* genomic DNA was obtained by phenol/chloroform extraction from sporozoites. Specific primers were obtained according to *E. tenella* His4 promoter sequence as previously described (7). PCR products of the last round were recovered and cloned into pEASY-T1-simple vector (TransGen Biotech), sequenced, and the results were analyzed by DNAStar 7.0 software. Twelve insertion site sequencing were conducted. Second, we applied Western blot to identify the “self-cleaving” effectiveness of P2A in transgenic parasites. Whole parasite soluble extracts were prepared from sporozoites as previously described (19). The lysis was centrifuged at 10,000 rpm for 10 min, and the supernatant was then subjected to SDS-PAGE and Western blot. Monoclonal antibody against His-tag (ABclonal) was applied. *E. tenella* GAPDH polyclonal antibody obtained and preserved in our lab was included as a loading control to ensure similar amounts of parasites’ soluble antigen were applied.

### Observation on Whole Life Cycle of *EmagER*

Seven 4-week-old rabbits were orally inoculated with sporulated oocysts of *EmagER* from the fifth passage and euthanized at 24, 48, 72, 84, 120, 144, and 152 h post inoculation (hpi) (*n* = 1 animal per time point) as described before (13). Jejunum and ileum were washed with cold HBSS, and smears were made by scraping the mucosa of the intestine. Fresh smears and sporulation process of newly collected oocysts were visualized under a confocal laser scanning microscopy (SP5, Leica, Germany) for observation of different developmental stages of fluorescent parasites.

### Reproductivity and Immunogenicity of *EmagER*

To explore the biological features of the transgenic strain, reproductivity and immunogenicity compared with the parental strain (*EmagWT*) were investigated. Sixteen 40-day-old rabbits housed one per cage were randomly distributed into four groups with four rabbits per group. Two groups of rabbits were orally inoculated either with 200 oocysts of *EmagWT* or *EmagER*. The third group was not immunized and applied as an unimmunized challenged control. The fourth group was applied as an unimmunized and unchallenged control (UUC). Daily oocyst outputs during patent period (6.5–12 dpi) of each infected rabbit were counted according to a standard McMaster technique (18). Also, 14 dpi, a challenge with 1 × 10^4^ sporulated oocysts of *EmagWT* was performed to each rabbit except for the ones in the UUC group. Oocyst outputs of all challenged rabbits were counted. Clinical signs of experimental animals were monitored every day, and body weight was measured twice a week during the whole experimental process.

### Immunization and Real-time PCR

To investigate the exogenous protein-specific immunity stimulated by the transgenic parasites, fifteen 35-day-old coccidia-free rabbits were randomly distributed into three groups. Two groups of rabbits were inoculated with 200 oocysts of either *EmagWT* or *EmagER*. The last group of rabbits was inoculated with 200 µl PBS as control. Fourteen days later, all rabbits were euthanized. Splenocytes and mesenteric lymph nodes (MLN) lymphocytes were isolated, and single cell suspensions were prepared. Lymphocytes were adjusted to 5 × 10^6^/ml and grown in RPMI-1640 culture medium supplemented with fetal bovine serum [10% (v/v)], penicillin (200 U/ml)–streptomycin (streptomycin) at 37°C in an atmosphere of 5% CO_2_. *EmagWT*-soluble antigen (EmagSA) (20 µg/ml), recombinant EYFP (rEYFP) (20 µg/ml), and recombinant RFP (rRFP) (20 µg/ml) were added separately as *in vitro* stimulus. A cell stimulation cocktail containing PMA (40.5 µM) and ionomycin (670 µM) (eBioscience) was used as a positive stimulus. Lymphocytes were harvested 12 h after stimulation, washed twice with sterilized PBS, and total RNA was extracted using TRIzol reagent (Invitrogen, Shanghai). Total cDNA was synthesized using an EasyScript First-Strand cDNA Synthesis SuperMix (Transgen, China). The primer pairs used for analysis of four specific genes (Table 1) were designed through NCBI Primer Designing Tool (http://www.ncbi.nlm.nih.gov/tools/primer-blast/). Quantitative real-time PCR was performed using a SYBR^®^ Premix Ex Taq™ (Takara) on 7500 Real-Time PCR System (Applied Biosystems) with a program of 50°C for 2 min, 95°C for 10 min, 40 cycles of 95°C for 15 s, and 60°C for 1 min. Relative gene expression was calculated by the 2^−ΔΔCq^ (Livak) method (20).

**Table 1:** **Primer pairs for quantitative real-time PCR**.

Target gene	Forward and reverse primer sequences 5′–3′	Target size (bp)	NCBI accession
GAPDH	F: CGAGACACGATGGTGAAGGT	166	NM_001082253.1
	R: TGCCGTGGGTGGAATCATAC		
IFN-γ	F: GCTCTGCCTCATCTTGGGTT	117	NM_001081991.1
	R: GGTCCACCATTTGCCACATC		
IL-2	F: GCCCAAGAAGGTCACAGAATTG	128	NM_001163180
	R: TGCTGATTGATTCTCTGGTATTTCC		
TNF-α	F: AGCCCACGTAGTAGCAAACC	192	NM_001082263.1
	R: TGAGTGAGGAGCACGTAGGA		

*Primer pairs were designed within the coding sequences (CDS) of rabbit genes available at NCBI. All primers were designed using Primer-BLAST and made to span exon junctions when possible*.

### Statistical Analysis

Statistical analysis was performed by one-way ANOVA using the SPSS 19.0 software. Data was presented as mean ± SEM. Difference between groups were considered statistically significant when *p* values were less than 0.05.

## Results

### Construction and Identification of a Transgenic Line of *E. Magna* Expressing EYFP and RFP (*EmagER*)

For *in vitro* transfection, fluorescent sporozoites in MDBK cell culture were observed 20 h after inoculation (Figure 1B). As the foreign genes were initiated by His4–NLS, EYFP was mainly expressed in the nuclei of the sporozoites. Meanwhile, RFP was found both in the nuclei and the cytoplasm indicating that the transfected plasmid can be employed by *E. magna*. After several trials on *in vivo* transfection, we obtained a transgenic population at a transfection efficiency of 0.06%. The fluorescent rate increased to 40% after five cycles of FACS and propagation in coccidia-free rabbits. Unfortunately, we did not obtain higher fluorescent rate in subsequent passages.

All transgenic oocysts express EYFP in the nuclei and RFP both in the nuclei and cytoplasm (Figure 1C). To further determine the function of P2A in *EmagER*, Western blot using an mAb against His-tag, which was ligated to RFP in the transfection plasmid, was performed. A 26 kDa (RFP-6 × His) instead of a 128 kDa (DHFR-EYFP-RFP-6 × His) band was detected demonstrating that P2A was efficient as self-cleaving peptide in *E. magna* (Figure 1D). Genome walking analysis of the oocysts from fifth passages were conducted (Figure 1E), and the 5′ integration site was determined (Figure 1F). Collectively, we obtained a transgenic *E. magna* strain expressing EYFP and RFP.

### *EmagER* Expresses Exogenous Proteins Targeted to Different Cellular Compartment throughout the Entire Life Cycle

**Figure 2:**
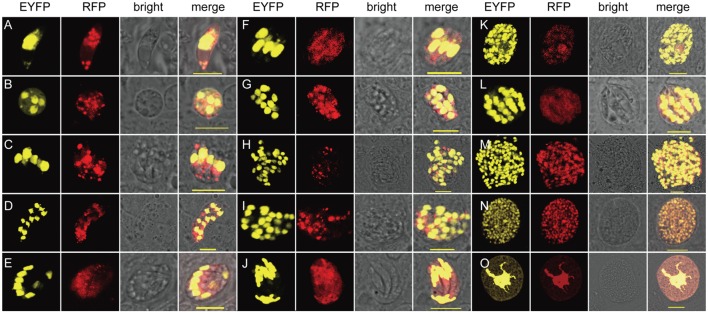
**Exogenous proteins were expressed and targeted to different cellular compartments in endogenous developmental stages of *EmagER***. **(A)** A sporozoite at 24 hpi; **(B)** a trophozoite at 24 hpi; **(C–M)** first to fourth generations of meronts of both type A (multinucleate) and type B (uninucleate) meronts at 48–120 hpi; **(N)** a microgametocyte at 152 hpi; and **(O)** a macrogametocyte at 144 hpi; bar = 20 μm.

By taking advantages of the fluorescent proteins expressed by the transgenic parasites, the morphological features of transgenic parasites during all developmental stages were observed (Figure 2). Invading sporozoites as well as trophozoites ongoing nucleus division were found at 24 hpi (Figures 2A,B). Mature meronts of each generation were found at 48, 66, 102, and 120 hpi, respectively (Figures 2C–M). Since EYFP was mainly expressed in the nuclei, both multinucleate and uninucleate merozoites were vividly distinguishable. Mature microgametocytes containing thousands of microgametes (Figure 2N) and macrogametocytes whose EYFP was not only located within the large nucleus but also Golgi adjunct (21) (Figure 2O) were discovered during 144–152 hpi. During the whole endogenous stages, the transgenic parasites express EYFP in the nucleus and RFP in the nucleus and cytoplasm.

**Figure 3:**
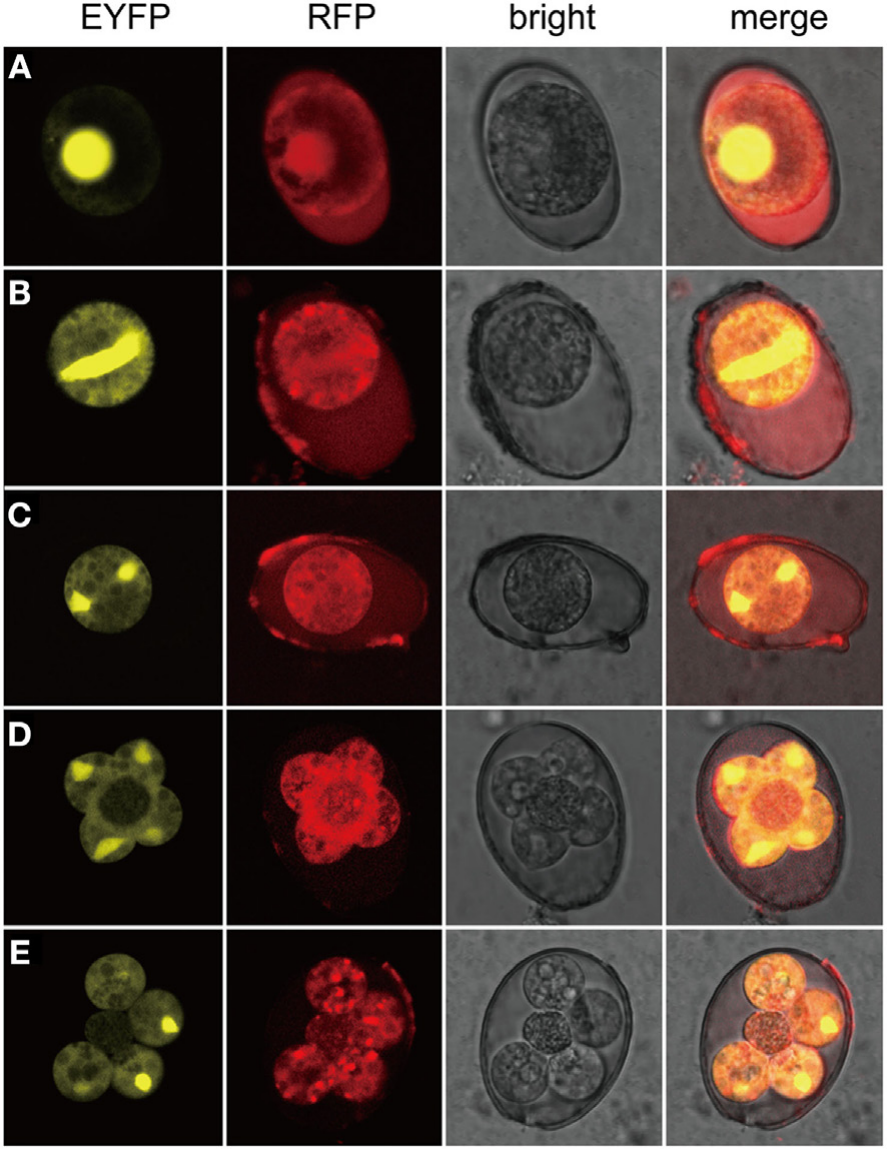
**Expression of exogenous proteins of *EmagER* during sporogony**. Freshly collected oocysts of *EmagER* were incubated in 2.5% potassium dichromate at 27°C and observed at different time intervals under a confocal microscope (Leica, SP5, Germany). **(A)** A freshly collected unsporulated oocyst; **(B)** an oocyst during the process of spindle stage; **(C)** first nuclear division finished; **(D)** four separated sporoblasts projected from the central cytoplasmic mass during the second nuclear division; and **(E)** formation of four spheres of sporocysts and the oocystic residua.

In addition, sporogony of the transgenic parasites was also observed (Figure 3). Oocysts of the transgenic strain, freshly collected from fecal samples and purified by floatation with saturated salt water were applied (Figure 3A). The first nuclear division proceeded after contraction or shrinkage of the cytoplasmic mass (Figures 3B,C). Completion of the second nuclear division and cytokinesis was evidenced by four sporoblasts symmetrically projected from the central cytoplasmic mass (Figure 3D) and soon developed into four separated spheres and an oocystic residua (Figure 3E). During all three nuclear division and two cytokinesis, EYFP was located in the nucleus and RFP mainly in the cytoplasm (Figure 3). Observation of all the developmental stages of the transgenic parasites demonstrated that the exogenous proteins were expressed and targeted to different cellular compartments.

### Reproductivity and Immunogenicity of *EmagER*

**Figure 4:**
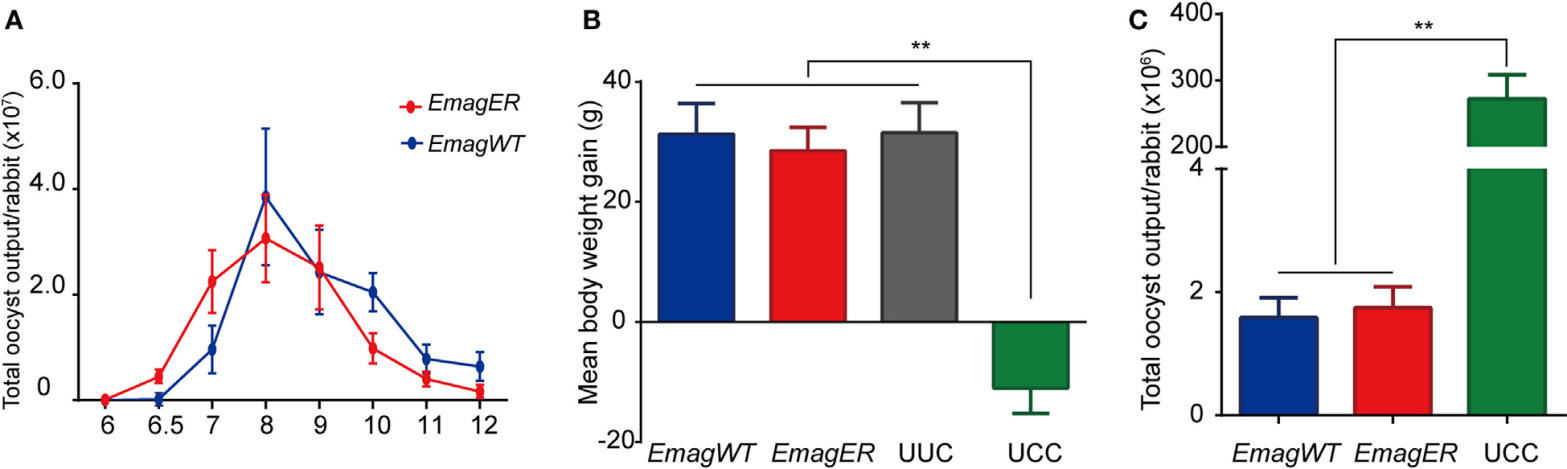
**Reproductivity and immunogenicity of *EmagER***. Three groups of rabbits were inoculated with either 200 oocysts of *Eimeria magna* wild-type (*EmagWT*), transgenic *E. magna-*expressing enhanced yellow fluorescent protein and red fluorescent protein (*Em*ag*ER*), or 200 µl PBS, respectively. **(A)** Daily oocyst output of individual rabbit during patent period were measured using a McMaster counting chamber. Fourteen days later, a challenge of 1 × 10^4^ oocysts of *EmagWT* was performed to all rabbits. Body weight gain **(B)** and total oocyst output **(C)** were measured in 14 days post challenge. UUC, unimmunized and unchallenged control; UCC, unimmunized and challenged control. All data were presented as mean ± SEM values. Statistical analysis was performed by one-way ANOVA, LSD, *p < 0.05, and **p < 0.01.

To investigate the biological feature of *EmagER*, reproductivity and immunogenicity were evaluated. First, daily oocyst outputs of rabbits infected with either *EmagWT* (200 oocysts) or *EmagER* (200 oocysts) were measured 6–12 dpi. Oocysts of both strains were first shed on 6.5 dpi, increased sharply, and reached the peak on 8 dpi (Figure 4A). *EmagER* presented slightly reduced reproductivity (mean ± SEM, 9.464 × 10^7^ ± 1.275 × 10^7^) compared with *EmagWT* (mean ± SEM, 1.057 × 10^8^ ± 2.244 × 10^7^). And no significant difference was found (one-way ANOVA, LSD). Next, a challenge of 1 × 10^4^ oocysts of *EmagWT* was performed to all rabbits 14 dpi in order to evaluate immunogenicity of the transgenic parasites. Robust immunity was evidenced, and no significant difference of body weight gain and oocyst reduction were observed compared with *EmagWT* (Figures 4B,C). Collectively, *EmagER* was of equal reproductivity and similar immunogenicity as *EmagWT*.

### Exogenous Proteins Expressed by *EmagER* Stimulated Local Immune Response

**Figure 5:**
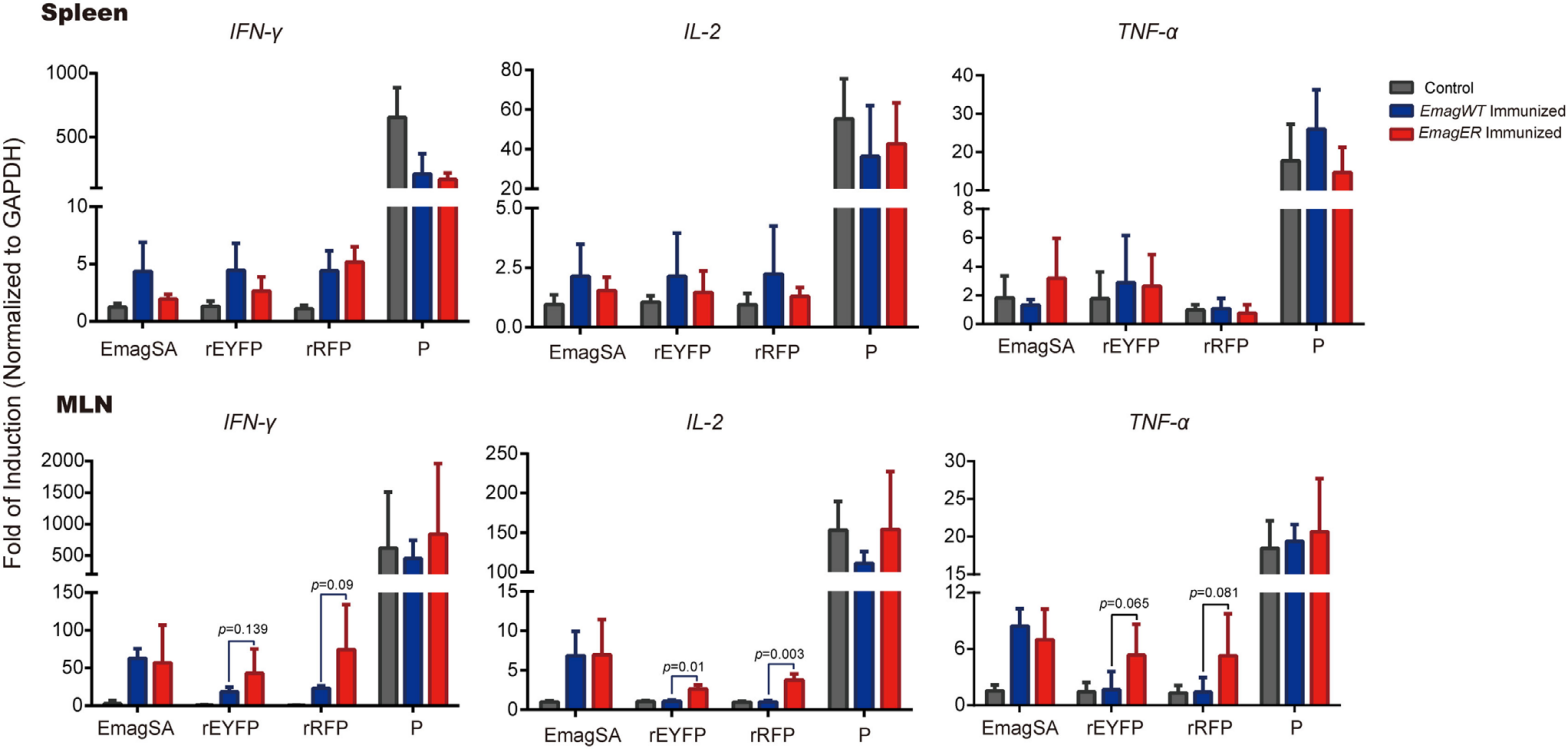
**Transcriptional levels of Th1 cytokines of splenocytes and mesenteric lymph nodes (MLN) of rabbits immunized with *EmagWT* and *EmagER* after *in vitro* stimulation**. Three groups of rabbits were inoculated with either 200 oocysts of *EmagWT, Em*ag*ER*, or 200 µl PBS, respectively. Splenocytes and MLN lymphocytes were isolated and stimulated with 20 µg/ml of either EmagWT-soluble antigen, recombinant enhanced yellow fluorescent protein, recombinant red fluorescent protein, or positive stimulus (P, containing 40.5 µM PMA and 670 µM ionomycin). Quantitative real-time PCR was performed using GAPDH as an internal control. Relative gene expression was calculated by the 2^−ΔΔCq^ (Livak) method. All data were presented as mean ± SEM values. Statistical analysis was performed by one-way ANOVA, LSD.

The Th1 cytokine profile is a well-known indicator of cellular immunity elicited by intracellular pathogens including *Eimeria* spp. (22). To determine whether the exogenous proteins expressed by the transgenic parasites can stimulate specific immunity, we investigated the mRNA transcriptional level of Th1 cytokines of lymphocytes of immunized rabbits after an *in vitro* stimulation. Splenocytes and lymphocytes of MLN of experimental rabbits were isolated, stimulated, and mRNA transcriptional level of IFN-γ, IL-2, and TNF-α was analyzed by real-time PCR (primers were shown in Table 1). We found that the transcriptional level of IFN-γ, IL-2, and TNF-α of the splenocytes was relatively low, and there was no significant difference among immunized or non-immunized rabbits after *in vitro* stimulation (Figure 5). In MLN lymphocytes, on the other hand, EmagSA stimulation caused upregulated Th1 cytokine transcription of *EmagWT-* and *EmagER*-immunized rabbits. Furthermore, relative expression level of IFN-γ, IL-2, and TNF-α after stimulation with rEYFP and rRFP was higher in *EmagER-*immunized rabbits than that of other groups (Figure 5). This result indicated that *EmagER* stimulated exogenous protein-specific local immune response in rabbit.

## Discussion

The transgenesis of parasites in the phylum Apicomplexa has made great progress in recent years. In chickens, at least two (*E. mitis, E. tenella*) of the seven most important *Eimeria* spp. have been transfected (6, 7). Much more detailed work in this area has been done with *T. gondii* (23), *Plasmodium* spp. (24), and *Cryptosporidium* spp. (25). In rabbits, however, the only work done on transgenesis was developed by Shi et al. (26) in a strain of *Eimeria intestinalis*. Here we report, for the first time, the construction of a transgenic line of *E. magna*, from the domestic rabbit, which expresses the dual reporter genes, EYFP and RFP. More importantly, the exogenous proteins expressed by this transgenic parasite stimulated a specific local immune response in our rabbits. This discovery in rabbits offers the prospect that transgenic rabbit coccidia might be candidates to transport other proteins as recombinant biological vaccines.

Here, we used the regulatory sequences of *E. tenella*, which was shown to be reliable in a previous study on transfection of *E. intestinalis* (26) in a plasmid generated by Tang et al. (10). This reliability might result from the functional conservation of *E. tenella* histone 4 and actin genes among these *Eimeria* species. Additionally, Tang et al. (10) provided an in-depth study on *E. tenella* demonstrating the “self-cleavage” peptide 2A from porcine teschovirus-1. We also found that it was efficient in our transgenic *E. magna*. The regulator sequences and the “self-cleavage” peptide are simple, and efficient molecular tools may promote additional studies that will be useful in developing transgenic models with *Eimeria* species in any species.

As noted earlier, successful transfection studies have been accomplished with two species of chicken coccidia. Liu et al. (15) achieved 0.2% transfection rate with *E. tenella*. Later, Qin et al. (7) showed a similar rate (0.19%) for *E. mitis*. The first study on transfection with rabbit coccidia by Shi et al. (26) involved *E. intestinalis*, but they succeed only 0.01% in their first passage. This lower transfection rate might be due to a higher susceptibility of mammalian sporozoites to electroporation than are those of bird coccidia for some, as yet, unknown reasons. In our study, the transfection rate in our first passage of *E. magna* was similar (0.06%). Unfortunately, this is where the similarity ends between these two rabbit *Eimeria* spp. Shi et al. (26) demonstrated that the transgenic parasites grew up to 80% in the third passage by selection of FACS and propagation, whereas we obtained only 40% fluorescent parasites even with a system of DHFR-Ts2m3m after five passages. As the plasmid was randomly integrated into the parasite’s genome, the site of integration may affect the transcription rate of the gene of interest (27). Unfortunately, we cannot confirm this due to the lack of an annotated reference genomic library.

In spite of what some might perceive as relatively low transfection success, our constitutive expression of fluorescent proteins presented major advantages that allowed us to identify developmental stages of whole life cycle of *E. magna*. Similar findings also were stated in research on transgenic *E. mitis* (7) in chickens and *E. intestinalis* (26) in rabbits. The major advantage of our procedures, however, is that we transfected dual reporter genes that targeted different cellular compartments. That is, EYFP is expressed mainly in the nuclei of all developmental stages of *E. magna* and the RFP targeted to the cytoplasm. Subcellular localization of RFP in *EmagER* was different from a previous study on *E. tenella* where RFP was also regulated by gran8 yet secreted into the parasitophorous vacuole (17). Nevertheless, in the future, we could engineer other proteins, using the same vector, to deliver new antigens into the organelles of the various endogenous stages of rabbit coccidia, and perhaps other coccidia of domesticated mammals.

We did not observe significant systemic immune response as measured by cultured splenocytes, probably due to the low sensitivity of the test. However, a significant local immunity in the MLN was detected. Previous work in our laboratory (8) on transgenic chicken *Eimeria* demonstrated that enhanced cellular immunity was detected in PBMC of chickens immunized with transgenic parasites expressing chicken IL-2. However, the systemic immune response, at least in our rabbits, was low after primary infection of *EmagWT* or *EmagER*, consistent with a previous study on the dynamics of T-lymphocytes in rabbits immunized with *E. intestinalis* (28). In fact, enhanced transcriptional levels of Th1 cytokines in MLN, after stimulation with EmagSA in immunized rabbits, indicated that protection against *E. magna* was established due to an effective local immune response. In particular, the exogenous proteins expressed by our transgenic *E. magna* induced cellular immunity in MLN as well. Our findings confirmed the capacity of transgenic rabbit coccidia to introduce foreign antigens into the rabbit immune system and showed the prospect of transgenic rabbit coccidia as a live vaccine vector.

## Author Contributions

XS, GT, and TS designed this study. GT and TS carried out the experiments with the help of XMT, YW, CL, JS, XLT, and XL. XS supervised the study implementation. GT drafted the manuscript. TS and DD contributed to the revision of the manuscript. All the authors read and approved the final version of the manuscript.

## Conflict of Interest Statement

The authors declare that the research was conducted in the absence of any commercial or financial relationships that could be construed as a potential conflict of interest.
